# Advanced practice registered nurses, physician assistants and cancer prevention and screening: a systematic review

**DOI:** 10.1186/1472-6963-14-68

**Published:** 2014-02-12

**Authors:** Alexandria A Smith, Deanna Kepka, K Robin Yabroff

**Affiliations:** 1 American Legacy Foundation, Washington, DC, USA; 2 Division of Cancer Control and Population Sciences, National Cancer Institute, Bethesda, MD, USA; 3 College of Nursing, University of Utah, Salt Lake City, UT; 4 Cancer Control and Population Sciences, Huntsman Cancer Institute, Salt Lake City, UT

## Abstract

**Background:**

For more than two decades, integration of team-based approaches in primary care, including physicians, advanced practice registered nurses and physician assistants (APRN/PA), have been recommended for improving healthcare delivery, yet little is known about their roles in cancer screening and prevention. This study aims to review the current literature on the participation and roles of APRN/PAs in providing cancer screening and prevention recommendations in primary care settings in the United States.

**Methods:**

We searched MEDLINE and CINAHL to identify studies published in 1990–2011 reporting on cervical, breast, and colorectal cancer screening and smoking cessation, diet, and physical activity recommendations by APRN/PAs in the United States. A total of 15 studies met all of our eligibility criteria. Key study, provider, and patient characteristics were abstracted as were findings about APRN/PA recommendations for screening and prevention.

**Results:**

Most studies were cross-sectional, showed results from within a single city or state, had relatively small sample sizes, reported non-standardized outcome measures. Few studies reported any patient characteristics. APRN/PAs are involved in recommending cancer screening and prevention, although we found variation across screening tests and health behavior recommendations.

**Conclusions:**

Additional research on the cancer prevention and screening practices of APRN/PAs in primary care settings using standardized outcome measures in relation to evidence-based guidelines may help strengthen primary care delivery in the United States.

## Background

In the United States (US), cancer is the second leading cause of death [1]. According to current US Preventative Services Task Force (USPSTF) practice guidelines, cancer screening is associated with reduced cervical, breast, and colorectal cancer mortality and efforts to promote tobacco cessation [2-4], a healthful diet, and increased physical activity are associated with reduced cancer risk [5]. Yet in some populations with historically poor cancer outcomes, particularly the uninsured, low-income, and minorities [6,7], these evidence-based cancer control interventions have not been fully adopted [8]. The Affordable Care Act will give approximately 32 million Americans greater access to healthcare and specifically the aforementioned cancer screening and prevention methods without any copayment by 2014 [9,10].

A recent Institute of Medicine Report (2011) called upon nurses to help meet the goals of the Affordable Care Act and outlined state and federal policy strategies to help ensure that nurses practice to the full extent of their education and training. For example, because APRN/PAs are not consistently able to see patients and provide medications without a physician’s supervision, the report calls for reform of states’ scope-of-practice laws. Furthermore, this report calls on nurses to serve as full partners with physicians and other healthcare professionals in the redesign of the US healthcare system and provides educational opportunities [11]. In addition, the Affordable Care Act provides for the Expansion of Physician Assistant Training Program [12], aims to increase student enrollment in primary care Physician Assistant (PA) programs. Since 1990 the number of APRN/PAs working in the US has risen from 50,000 to 250,000 in 2010, with midlevel providers conducting an increasing role in serving underserved populations and locations [13,14]. Approximately 55% APRNs/PAs work in a primary care setting [15]. The US Bureau of Labor Statistics reports that physician assistants within the United States are expected to grow by 30% between 2010 and 2020 [16]. Thus, this growing cadre of primary care health professionals is uniquely positioned to meet the growing demand for primary care resulting from the expansion of healthcare coverage by the Affordable Care Act.

As more individuals enter into the health care system, widespread implementation of evidence-based cancer screening and prevention interventions is critical for reducing cancer mortality and morbidity, but taxing on an already burdened healthcare system. In order to fulfill all of the current US Preventative Services Task Force recommendations for an average size patient panel, a primary care provider would exhaust an estimated 7.4 hours each day before providing diagnoses, treatments, or conducting administrative tasks [17]. Advanced practice nurses and physician assistants (APRN/PAs), including Nurse Practitioners (NPs), Physicians Assistants (PAs), and Certified Nurse Midwives (CNMs) are positioned to help meet this demand for recommended preventive services. For more than two decades, recommendations have been made to include APRN/PAs within primary care teams to improve chronic care delivery systems [18-21]. However, little is known about the roles of APRN/PAs in cancer screening and prevention, or how they might help meet an increasing demand for care. In this study, we conducted a systematic review of the recently published literature to assess the recommendations for and provision of USPSTF recommended cancer prevention and screening by APRN/PAs in primary care settings.

## Methods

### Study selection

We used the PubMed search mechanism for MEDLINE and CINAHL to identify English language studies on USPSTF recommended cancer prevention and screening recommendations among APRN/PAs in the United States published between January 1990 and December 2011. The search strategy used a combination of NIH librarian recommended terms that addressed APRN/PAs, cervical, breast, or colorectal cancer screening or recommendations for smoking cessation, or diet and physical activity. Using the following combination of search terms, a total of 594 studies were found: “Early Detection of Cancer” or “Vaginal Smears” or “Mammography” or “Breast Neoplasms/prevention and control” or “Colorectal Neoplasms/prevention and control” or “Mass Screening” or “Papillomavirus Vaccines” and “Diet” or “Exercise” or “Motor Activity” or “Tobacco Use Cessation” and “Counseling” or “Nurse’s Role” or “Preventive Health Services” or “Patient Acceptance of Health Care” or “Patient Education as Topic” or “Health Knowledge, Attitudes, Practice” and “Nurse Practitioners” or “Physician Assistants” or “Nurse Midwives”. Organization of search terms and the number of published studies identified with each set of terms can be found in Table 1.

**Table 1:** Search terms used in med line and CINAHL

**Group number**	**Search term**	**Number of articles medline**	**Number of articles CINAHL**
Group 1	Nurse Practitioners OR	22,644	3,779
	Physician Assistants OR		
	Nurse Midwives		
Group 2	“Counseling”[Mesh:noexp] OR	576,608	9,166
	“Nurse’s Role”[Mesh] OR		
	“Patient Acceptance of Health Care”[Mesh] OR		
	“Patient Education as Topic”[Mesh] OR		
	“Health Knowledge, Attitudes, Practice”[Mesh]		
Group 3	“Diet”[Mesh] OR	335,235	52,213
	“Exercise”[Mesh] OR		
	“Motor Activity”[Mesh] OR		
	“Tobacco Use Cessation”[Mesh]		
Group 4	“Early Detection of Cancer”[Mesh] OR	132,489	4,199
	“Vaginal Smears”[Mesh] OR		
	“Mammography”[Mesh] OR		
	“Breast Neoplasms/prevention and control”[Majr:noexp] OR		
	“Colorectal Neoplasms/prevention and control”[Majr:noexp] OR		
	“Mass Screening”[Mesh] OR		
	“Papillomavirus Vaccines”[Mesh]		
	Group 1 and Group 2 and (Group 3 or Group 4)	573	21

Limits: Humans, Journal Article, English, Publication Date from 1990/01/01 to 2011/12/31.

Abstracts were reviewed to identify the type of cancer screening or prevention, study design, sample size, and the country where the study was conducted. Inclusion criteria for this review were studies reporting: APRN/PAs conducting cancer prevention screening for cervical, breast or colorectal cancer, recommendations for smoking cessation, or diet and physical activity; United States primary care setting; study publication dates between January 1990 and December 2011, quantitative data and at least 100 participants. We used a sample size eligibility requirement to ensure stable estimates of cancer prevention and screening as has been done [22,23]. Only 4 studies of more than 500 studies were excluded based solely on sample size. Reviews, editorials, letters, and essays were also excluded. Articles were initially reviewed by one author and any further decisions were discussed by all authors to determine if the study met all the eligibility criteria.

Ten studies met all of our eligibility criteria [24-33]. Because electronic searches may not identify all relevant studies [34], reference lists of the selected studies and published reviews of APRN/PAs and cancer screening or prevention were reviewed to identify other eligible studies. Five additional studies were identified in this manner [35-39]. A total of 15 studies are included in this paper.

### Data abstraction

Data were abstracted from each paper using a standardized format in an excel spreadsheet. *Study characteristics included:* type of cancer screening test (Papanicolaou/Pap test, mammogram and/or any colorectal cancer screening test, including fecal occult blood test (FOBT), flexible sigmoidoscopy, or colonoscopy) or prevention recommendations (smoking cessation, diet and/or physical activity), geographic setting of sample (national, state, local), study design (cross-sectional, intervention and retrospective cohort), study size, delivery setting (single institution, network of institutions and multiple non-network institutions) and year of publication. *Type of APRN/PA* included NPs, CNMs, and PAs and was not a mutually exclusive category because some studies combined multiple types of APRN/PAs. Due to this and the limited number of studies, we reported on APRN/PAs as a combined unit unless the studies only focused on type of provider. The *comparison group* was recorded in three categories: physicians, other provider, and no comparison group. Where possible, comparisons were made between physicians and APRN/PAs. The *patient and provider characteristics* measured were age, gender, and race/ethnicity. The types of patient insurance coverage were also abstracted when reported. Cancer screening and prevention recommendations findings were abstracted as reported. Because some studies evaluated multiple screening tests and multiple types of prevention, these findings were abstracted and reported separately. The study cancer screening and prevention *outcome measures* were recorded in three categories: self-report, chart review, and biological samples. All study findings were abstracted as reported in the underlying study. This study fully conforms to the PRISMA guidelines for systematic reviews (http://www.prisma-statement.org).

## Results

### Study characteristics

A total of seven studies reported outcomes on screening for cervical, breast or colorectal cancer [24,26,28-31,39], while ten studies measured cancer prevention recommendations for smoking cessation, diet, and physical activity (Table 2) [25-27,31-33,35-38]. We did not identify any studies of HPV vaccination or of post-treatment survivorship care. Three studies reported outcomes on more than one type of cancer screening [24,26,39], and four studies reported on more than one type of cancer prevention recommendations [26,27,31,37]. Most studies were cross-sectional, showed results from within a single city or state, had relatively small sample sizes (less than 500) and reported on the behavior of NPs. Only three studies reported on interventions, two focusing on tobacco and one on Pap test and mammograms [32,38,39]. The majority of studies presented self-reported data from providers about their own practice or their perceptions of APRN/PAs practice [24,25,28,31,35,37], while only a few presented self-reported data from patients [32,38]. Few studies reported receipt of services or documented changes in behavior as part of chart reviews [26,27,36,39]. A small number reported any patient characteristics. The response rates for studies varied, with those that presented self-reported data from providers ranged from 30% to 72% [24,25,28-31,35,37]. Many of the studies did not specify whether the APRN/PAs provided or recommended cancer screening.

**Table 2:** Characteristics of studies of Advanced Practices Registered Nurses and Physician Assistants (APRN/PA) and cancer prevention and screening

**Study characteristics**	**Study categories**	**Number of studies (N = 15)**	**Percentage of studies**
Type of cancer prevention*			
	Diet/Physical activity	5	33%
	Smoking	9	60%
	Mammogram	3	20%
	Pap test	5	33%
	Colorectal cancer screening	4	27%
Study setting			
	National	6	40%
	State or regional	5	33%
	Local (City or Multiple Counties)	4	27%
Study design			
	Intervention	3	20%
	Retrospective cohort	2	13%
	Cross sectional	10	67%
Study date of publication			
	<1995	2	13%
	1995-1999	3	20%
	2000-2004	4	27%
	> = 2005	6	40%
Type of APRN/PA provider			
	Advanced practice nurse practitioner	13	87%
	Certified nurse midwife	2	13%
	Physician Assistant	3	20%
Comparison group			
	Physicians	5	33%
	None	5	33%
	Other	5	33%
Sample size			
	100-499	8	53%
	500-999	1	7%
	1,000-4,999	5	33%
	5,000-19,999	0	0%
	>20,000	1	7%
Healthcare delivery setting			
	Single institution or clinic	2	13%
	Network of institutions or clinics	3	20%
	Multiple institutions (Not a Network)	10	67%
Outcome measure			
	Chart review	3	20%
	Self-Report by physicians	4	27%
	Self-Report by APRN/PA providers	6	47%
	Self-Report by patients	2	13%
Patient characteristics			
Insurance types*			
	Any medicare	2	13%
	Any medicaid	5	33%
	Private	6	40%
	Not reported	8	60%

*Measures not mutually exclusive.

### Cancer screening

#### Cervical cancer screening

Of the five studies evaluating Pap tests, most showed that APRN/PAs provide or recommend Pap tests to patients (72% to 98%) and that physicians who currently work with APRN/PAs are amenable to APRN/PAs conducting Pap tests (Table 3) [24,26,29,31]. Physicians who practice in provider teams that include NPs and PAs are more supportive of NPs and PAs performing Pap tests that physicians who do not practice in provider teams that include NPs and PAs [29]. In addition, an intervention study compared NPs recommending and performing cervical cancer screening during routine visits to a provider reminder system. At follow-up there was a significant increase in the annual rate of women screened for cervical cancer by a NP at the intervention location (from 17.8% to 56.9%), while the annual rate of screening by physicians at the control location improved less (from 11.8% to 18.2%) during the study time period [39].

**Table 3:** Studies of Advanced Practices Registered Nurses and Physician Assistants (APRN/PA) and breast, cervical and colorectal cancer screening

**Study**	**Sample size**	**Outcome measure**	**Setting and study design**	**Pap findings**	**Mammogram findings**	**Colorectal cancer screening findings**
Menees et al., [24]	Total providers: 336 OB/GYNS: 182	Self-Report of MDs and NPs	National survey of OB/GYNs and NPs	Pap test routinely provided:	Mammography routinely recommended:	CRC screening routinely recommended:
				NPs: 94.8%	NPs: 90.9%	NPs: 61.7%
			Cross sectional Survey			
	NPs: 154			OB/GYNs: 97.8%	OB/GYNs: 98.9%	OB/GYNs: 87.2%
				*P < 0.01*	*P < 0.01*	*P < 0.01*
				Unadjusted	Unadjusted	Most common CRC screening recommended by either provider:
						FOBT: 76.2%
						Colonoscopy: 28.3%
						P-value not reported
						Ordered colonoscopy:
						NPs: 19.8%
						OB/GYNs: 37%
						P < 0.005
						All measures unadjusted
Hopkins et al., [26]	Total patients: 1339	Chart review	Chart review in private practice	Receipt of Pap test:	Receipt of mammogram: (Aged 40+)	Patient receipt of colorectal screening: (Ages 50 +)
	PHCC NP patients: 755		and primary care health centers in New York City	PHCC NPs: 71.5%	PHCC NPs: 69%	PHCC NPs: 19.1%
				PHCC MDs: 53.8%	PHCC MDs: 64.2%	PHCC MDs: 45.7%
	MD patients: 441		Retrospective cohort	P < 0.001	P = 0.240	P < 0.001
	PP NP patients: 143			Unadjusted	Unadjusted	Unadjusted
Sansbury et al., [28]	Total providers: 1900	Self-Report of MDs about APRN/PAs	National survey of MDs	NA	NA	Work with NP/PA to provide FOBT:
	PC MDs: 1235		Cross sectional survey			MDs report working with a NP or PA to provide FOBT: 23.8%
	NPC MDs: 665					
						Of the 24% of physicians who work with NP/PA for FOBT, they reported frequency of supervising a NP or PA for FOBT:
						Supervised a NP: 75%
						Supervised a PA: 25%
						P-value not reported for all measures
						All measures unadjusted
Oliveria et al., [29]	Total providers: 1363	Self-Report of MDs about APRN/PAs	National survey of MDs	MDs amenable to NP/PA screening:	NA	NA
	MDs: 1363		Cross sectional survey	Team Practice^: 89.6%		
				Non-team Practice^^: 59.9%		
				Team vs Non-team of amenable MDs:		
				OR = 8.11 (95% CI: 5.80–11.35)		
				P = 0.001		
				MDs reporting NP/PA screening:		
				All MDs reporting frequency of NPs or PAs performing Pap tests:		
				NPs: 33.5%		
				PAs: 23.2%		
				Team practice MDs reporting frequency of NP/PA performing Pap tests:		
				NPs: 89.3%		
				PAs: 82.7%		
				P-value not reported for all other measures		
				All measures unadjusted		
Shaheen et al., [30]	Total providers: 1784	Self-Report of APRN/PAs	Survey of NPs and PAs in North Carolina	NA	NA	NP/PA who recommend/perform FOBT:
	Total NPs: 526		Cross sectional survey			Primary Care PA: 94.6%
	Total PAs: 640					Primary Care NP: 92.1%
	PC PAs: 322					NP/PA who recommend/perform flexible sigmoidoscopy:
	PC NPs: 270					
						Primary Care PA: 76.1%
						Primary Care NP: 69.2%
						P-value not reported
						Unadjusted
Murphy, [31]	Total providers: 346	Self-Reportof CNMs	National survey of CNMs	98% of CNMs report they routinely provide pap tests to 81-100% of their gynecologic patients	NA	NA
	CNMs: 346		Cross sectional survey			
				P-value not reported		
				Unadjusted		
Mandelblatt et al., [39]	Total patients: 319	Chart review	Two New York City study hospitals with NP led intervention and usual care control	Receipt of Pap test in intervention group:	Receipt of mammography in intervention group:	NA
	Intervention: 160					
				Baseline: 17.8%	Baseline: 18.3%	
	Control: 159					
	Total providers: Not Reported					
			Intervention	Post: 56.9%	Post: 40%	
				P < 0.01	P < 0.01	
				Receipt of Pap test in control group:	Receipt of mammography in control group:	
				Baseline: 11.8%	Baseline: 18.1%	
				Post: 18.2%	Post: 18.2%	
				P-value not reported	P-value not reported	
				All measures unadjusted	All measures unadjusted	

^ = (MDs who work with NPs or PAs).

^^ = (MDs who do not work with NPs or PAs).

NA = Not Applicable.

NPC = Non-Primary Care.

NP/PA = NP or PA.

PHCC = Primary Health Care Center.

PC = Primary Care.

PP = Private practice.

* = A higher number means the provider does the behavior more frequently.

CI = Confidence Interval.

#### Breast cancer screening

Of the three studies that studied breast cancer, two showed that a majority of patients who see NPs receive mammograms (69% to 91%) and that NPs recommend a similar amount of mammograms as physicians (Table 3) [24,26]. In the same NP intervention study mentioned previously for cervical cancer screening, the annual rate of mammography screening increased more among women seen at the NP screening recommendation site (18.3% to 40.0%) than at the cancer screening program using a provider reminder checklist on charts and referrals (18.0% at both time points) [39].

#### Colorectal cancer screening

Findings about APRN/PAs involvement in colorectal cancer screening are mixed and vary based on the screening modalities evaluated (Table 3). Of the four colorectal cancer screening studies, three showed a range of reported colorectal cancer screening provided or recommended by APRN/PAs (19% to 95%) [24,26,30]. This large variation in reported colorectal cancer screening is partially determined by the variation in reporting amongst the studies. The lowest percentage is based on chart review of an unspecified type of colorectal cancer screening among patients aged 50 and above [26] while the highest percentage is a self-reported survey answer from providers on how often they recommend FOBT to any patient (age not specified) [30]. We also found substantial variation in reporting on colorectal cancer screening modalities, with some studies reporting specific modalities [24] and others reporting whether an unspecified type of colorectal cancer screening was offered [26]. In addition, two of the studies showed physicians reporting more colorectal cancer screening than APRN/PAs [24,26]. Only 24% of practicing primary care physicians reported working with APRN/PAs to provide FOBT in a nationally representative survey [28].

### Recommendations for cancer prevention

#### Smoking cessation recommendations

Both physicians and APRN/PAs report frequently providing smoking cessation recommendations (Table 4). APRN/PAs self-reported a range of assessment of tobacco use and smoking cessation recommendations to patients (7% to 95%) in eight studies [25,26,31,32,36]. Of the nine studies that measured tobacco related outcomes, three showed that patients are more likely to receive recommendations for smoking cessation during visits with NPs than during visits without NPs (associations not always statistically significant) while another study showed MDs feel that they are more adequately trained to give smoking cessation counseling than NPs [27,32,37,38]. One smoking cessation intervention of NPs at a prenatal clinic visit compared to a usual care control prenatal clinic visit reported significantly increased abstinence among cigarette smokers at follow-up (19% vs. 0%) [38].

**Table 4:** Studies of Advanced Practices Registered Nurses and Physician Assistants (APRN/PA) and diet, physical activity, and smoking cessation recommendations

**Study**	**Sample size**	**Outcome measure**	**Setting**	**Findings for diet**	**Findings for physical activity**	**Findings for smoking cessation**
Tompkins et al., [33]	Total providers: 398	Self-report of NPs	Survey of NPs at Pacific Northwest Annual National Conference	NA	Physical activity counseling of appropriate patient in past week:	NA
	NPs: 398					
			Cross sectional survey		25% of NPs reported counseling 50% of appropriate patients	
					37.75% of NPs reported counseling 75% of appropriate patients	
					14.8% of NPs reported counseling 100% of appropriate patients	
					Selected factors that facilitate physical activity counseling with patients:	
					69.2% of NPs reported length of patient visit	
					55.4% of NPs reported part of preventative health visit	
					P-value not reported	
Patton et al., [25]	Total providers: 1802	Self-report of MDs and NPs	Surveys of health professionals in North Carolina	NA	NA	NPs report that they assess:
						Patient’s past tobacco use: 95.1%
	Family physicians: 273					
						Patient’s present tobacco use: 97.9%
	NPs: 294					
	Dentists: 584		Cross sectional Survey			Type and amount of tobacco: 92.3%
	Hygienists: 651					
						P-value not reported
						Family MDs report that they assess:
						Patient’s past tobacco use: 98.5%
						Patient’s present tobacco use: 100%
						Type and amount of tobacco: 95.5%
						P-value not reported
						Adequately trained for smoking cessation
						NPs: 71.4%
						Family MD: 93.5%
						P-value not reported
						Physicians are significantly more likely to feel adequately trained to provide tobacco cessation compared to NPs
						OR = 5.3 (3.2 - 8.6)
						P-value<.0001
						All measures unadjusted
Price et al., [35]	Total providers: 194	Self-report of CNMs	Survey of CNMs in Ohio	NA	NA	CNMs reported that they always/usually:
	CNMs: 194		Cross sectional survey			Document cigarette smoking use status at each visit: 73%
	All patients pregnant women					Assess whether the patient is willing to make a quit attempt within the next 30 days: 66%
						Use counseling to help patients willing to make a quit attempt: 48%
						P-value not reported for all measures
						All measures unadjusted
Running et al., [36]	Total patients: 400	Chart review	Chart review of urgent care setting in HMO in the Southwest	NA	NA	Smoking cessation addressed among non-pharmacological interventions for sinusitis:
	NP patients: 200					
	Physician patients: 200					
			Retrospective cohort			NPs: 49%
						MDs: 31%
						Number of times smoking cessation is addressed for subjects in all categories
						NPs: .97
						MDs: 1.95
						P-value=.309
						Unadjusted
Hopkins et al., [26]	Total Patients: 1339	Chart review	Chart review in private practice and primary care health centers in NY City	Receipt of assessment and counseling on nutrition and diet:	Receipt of assessment and counseling on physical activity:	Receipt of assessment and counseling on tobacco use:
	Primary health care center (PHCC) NP patients: 755					
				PHCC NPs: 41.4%	PHCC NPs: 15.8%	PHCC NPs: 79.2%
				PHCC MDs: 14.7%	PHCC MDs: 2.5%	PHCC MDs: 87.8%
	MD patients: 441					
			Retrospective cohort	P-value=0.000	P-value=0.000	P-value=0.000
	Private practice NP patients: 143					
				Unadjusted	Unadjusted	Unadjusted
Lin et al., [27]	Total hospital outpatient department visits: 90,476	Chart review	National survey of hospital ambulatory settings (NAMCS)	Received diet counseling at an OPD visit with a NP compared to one without a NP	Received physical activity counseling at an OPD visit with a NP compared to one without a NP	Received tobacco use counseling at an OPD visit with a NP compared to one without a NP
	Visits with NP: 6,062		Cross sectional survey	32.6% vs. 22.9%	14.5% vs. 9.3%	6.7% vs. 4.3%
	Visits without NP: 84,416		Odds ratio adjusted for patient age, sex, clinic type, metropolitan status, geographic region of hospital, and number of providers seen.	Non-illness patients: 1.7 OR (95% CI OR: 1.2-2.5)	Non-illness patients: 1.8 OR (95% CI OR: 1.2-2.8)	Non-illness patients: 1.7 OR (95% CI OR: 1.2-2.5)
				P- value = 0.004	P- value = 0.007	P- value = 0.004
				OPD visits for patients with chronic problems with a NP compared to one without a NP:	OPD visits for patients with chronic problems with a NP compared to one without a NP:	OPD visits for patients with chronic problems with a NP compared to one without a NP:
				32.3% vs. 17.1%	20.2% vs. 8.9%	4.7% vs.2.9%
				2.5 OR (95% CI OR: 1.6-3.8)	2.8 OR (95% CI OR: 1.6-5.1)	1.8 OR (95% CI OR: 1.1-3.0)
				P-value = 0.001	P-value = 0.007	P-value = 0.01
Moody et al., [37]	Total Providers: 44	Self-report of	Survey of NPs in Tennessee	Provider report nutrition counseling:	Provider report physical activity counseling:	Provider report smoking cessation counseling:
	NPs: 44	NPs	Cross sectional survey	NPs: 19%	NPs: 12%	NPs: 7%
	Total patients: 680			MDs: 15%	MDs: 7%	MDs: 2.5%
				P-value not reported	P-value not reported	P-value not reported
				Unadjusted	Unadjusted	Unadjusted
Gebauer et al., [38]	Total patients: 178	Self-report of patients and Salivary Cotinine Sample	Follow up survey at outpatient obstetric clinic - state not specified	NA	NA	Smoking rate/day at follow-up: Mean (SD)
	Control patients: 94					Control: 13.7 (14.1)
	Intervention patients: 84		Intervention			Intervention: 7.8 (7.3)
	All patients pregnant women who report smoking and intervention includes being seen by an advance practice NP					P =.008
						Unadjusted
						Smoked any amount in past 7 days:
						Control Baseline: 94 participants
						Control Follow up: 94 participants
						Intervention Baseline: 83 participants
						Intervention Follow up: 70 participants
						Difference between groups =15.5% P-value<0.001
						Unadjusted
Murphy, [31]	Total providers: 346	Self-report of CNMs	National survey of CNMs	Nutritional counseling of gynecologic patients	Physical activity counseling of gynecologic patients	Smoking cessation counseling of gynecologic patients
	CNMs: 346		Cross sectional survey	52% of CNMs report counseling 81-100% of their patients	46% of CNMs report counseling 81-100% of their patients	72% of CNMs report counseling 81-100% of their patients
				P-value not reported	P-value not reported	P-value not reported
				Unadjusted	Unadjusted	Unadjusted
Zahnd et al., [32]	Total patients: 1217	Self-report of patients	Survey of patients from Four Kaiser Permanente Medical Centers in San Francisco Bay Area	NA	NA	Patients report discussing smoking cessation:
	NP patients: 269					NP Patients: 64%
	Physician patients: 948					MD Patients: 50%
						P-value<0.001
	Total providers: 52		Intervention			Unadjusted
	Physicians: 40					Independent predictors of counseling about smoking:
	NPs: 12					NP vs. Physician: OR 1.7
						P-value=.0006
						Adjusted for differences in patient characteristics

*=A higher number means the provider does the behavior more frequently.

CI = Confidence Interval.

NA = Not Applicable.

NPC = Non-Primary Care.

NP/PA = NP or PA.

PHCC = Primary Health Care Center.

PC = Primary Care.

PP = Private practice.

#### Diet and physical activity recommendations

The four studies that evaluated diet also evaluated physical activity, while one study only evaluated physical activity counseling (Table 4). These studies showed that while APRN/PAs do not frequently provide recommendations on diet and physical activity (12% to 52%), they do provide more recommendations related to diet and physical activity than their physician counterparts (3% to 15%) [26,27,31,37].

## Discussion

In this paper, we reviewed the recent literature on the participation and roles of APRN/PAs in the delivery of cancer prevention and screening recommendations in US primary care settings. In the descriptive or intervention research we identified, only 15 studies during a 21 year period, APRN/PAs are involved in recommending cancer screening and prevention. The limited research is somewhat surprising, because a team approach, including physicians and APRN/PAs, has long been recommended for improving healthcare [18-21,40]. After receiving the appropriate training, APRN/PAs expect to provide or recommend Pap tests, mammograms and FOBT, while studies only reported on physicians working concurrently with APRN/PAs to screen for cervical cancer [29] and colorectal cancer [28]. With the enactment of the Affordable Care Act, millions of previously uninsured or underinsured will gain access to healthcare. A better understanding of the potential roles of APRN/PAs in meeting this demand for cancer prevention and screening is critical.

The integration of more APRN/PAs into primary care can affect cancer screening and recommendations in several different ways. This integration has the potential to increase the overall percentage of the population ever receiving specific cancer prevention and screening recommendations, as was shown in an intervention study included in this review [39]. For example, colorectal cancer screening uptake in the US is substantially lower than for breast or cervical cancer screening [41]. The US Preventive Services Task Force (USPSTF) recommends any of three different tests for colorectal cancer (i.e., FOBT, flexible sigmoidoscopy, colonoscopy) [4]. These tests have different screening intervals, involvement of specialists, levels of invasiveness and other characteristics [4], potentially requiring detailed discussion to allow patients to make informed decisions about screening. Currently, less than 25% of physicians report actually working with APRN/PAs to provide colorectal cancer screening [28]. However, one challenge with moving forward with team-based health care is that physicians do not always want to work with nurse practitioners [42].

In a time constrained primary care setting, APRN/PAs might play a critical role in improving discussion about options and ultimately improving uptake of colorectal cancer screening. Alternatively, research featuring APRN/PAs might focus on improving all aspects of cancer control among specific populations, such as those previously uninsured or with key risk factors. Lack of health insurance and lack of prior screening has been consistently associated with late stage of disease at diagnosis for breast, cervical, and colorectal cancer [43-45]. Tobacco use and obesity are associated with many chronic diseases [46] and the role of APRN/PAs in encouraging healthy behaviors could improve a variety of health outcomes of the US population. Future research is needed that investigates that relationship between a visit with an APRN/PA and other primary care provider types within team-based primary care that oversamples racial and ethnic minorities and lower socioeconomic status populations.

We identified a number of methodological and reporting limitations in the studies included in this review related to study design and reporting of outcome measures and sample characteristics. Most of the studies were cross-sectional and did not assess cancer prevention or screening outcomes longitudinally. Surprisingly, only three studies reported results of interventions, therefore not allowing for a quantitative analysis of using APRN/PAs for cancer screening or prevention recommendations [32,38,39]. Few reported the type of APRN or PA provider separately, included comparison groups, or were based on well-described samples (Tables 3 and 4) [24-28,36,38,39]. In addition, studies that did include comparison groups did not consistently report on statistical significance of comparisons. Inconsistencies in outcome measure reporting among these studies impacted our ability to compare guideline adherence and patient populations. Few studies evaluated whether screening recommendations were consistent with evidence-based guidelines for patient age at initiation or frequency [24,26]. This is particularly important because both overuse and underuse of screening can have adverse patient outcomes [47-49]. Most of the studies neglected to report patient demographics or key covariates, such as weight, body mass index, and comorbidities, hindering our ability to determine if either physicians or APRN/PAs are providing cancer screening based on guidelines.

Outcome measures were most commonly reported using either provider or patient self-reported data about recommendations and did not report on receipt of service or a documented change in behavior [24,25,30-33,35,37]. Even further removed from receipt of service, some studies reported what physicians perceived of APRN/PAs practice [28,29]. Self-reported and proxy-reported data may over or underestimate documented receipt of APRN/PA provider services [50]. Further, primary care addresses multiple preventive services, but only about half of the studies included more than one aspect of cancer control and no studies address post-treatment survivorship care [24,26,27,31,37,39]. Future research should address these limitations and be conducted in longitudinal cohorts with comparison groups of well-described provider types, document patient receipt of screening or prevention recommendations, and assess multiple cancer control recommendations. Use of standardized measures, including for patient characteristics associated with guideline recommendations, evaluation of guideline adherence and longer term patient outcomes will also be important.

Despite using a large number of search terms to identify published studies, manually reviewing all abstracts and relevant reference lists, it is possible we missed some relevant studies. The studies we identified were fairly heterogeneous in terms of patient populations, geographic region, provider type, and type of a comparison group. Additionally, included studies used a variety of approaches to measure cancer screening and prevention, such as physician, non-physician provider and patient self-report, as well as chart review. As a result, our synthesis of findings was descriptive rather than quantitative. Findings are generalizable only to the primary care setting.

## Conclusion

In summary, further documentation of the role of APRN/PAs in recommending and providing cancer prevention and screening services in US primary care teams is needed. Ensuring that future research measures cancer screening according to evidence-based USPSTF guidelines decreases variability among measures reporting and focuses on receipt of services. This will allow stakeholders to make more informed decisions on how best to utilize this growing workforce and provide cancer prevention and screening services to the US population.

## Competing interests

The authors declare that they have no competing interests.

## Authors’ contributions

AS reviewed articles for initial eligibility, created tables and drafted manuscript. AS, DK and RY reviewed final articles for eligibility, provided study analysis and edited the manuscript. All authors read and approved the final manuscript.

## Pre-publication history

The pre-publication history for this paper can be accessed here:

http://www.biomedcentral.com/1472-6963/14/68/prepub
